# Antioxidant and Antiproliferative Activities of Methanolic Extract from a Neglected Agricultural Product: Corn Cobs

**DOI:** 10.3390/molecules19045360

**Published:** 2014-04-24

**Authors:** Raniere Fagundes Melo-Silveira, Gabriel Pereira Fidelis, Rony Lucas Silva Viana, Vinícius Campelo Soeiro, Rodrigo Augusto da Silva, Daisy Machado, Leandro Silva Costa, Carmen Veríssima Ferreira, Hugo Alexandre Oliveira Rocha

**Affiliations:** 1 Laboratório de Biotecnologia de Polímeros Naturais (BIOPOL), Departamento de Bioquímica, Centro de Biociências, Universidade Federal do Rio Grande do Norte (UFRN), Natal, Rio Grande do Norte, RN 59078-970, Brazil; E-Mails: ranierefagundes@hotmail.com (R.F.M-S.); gabrielfideliss@gmail.com (G.P.F.); rony_lucas@hotmail.com (R.L.S.V.); vihcampelo@gmail.com (V.C.S.); 2 Laboratório de Bioensaios *in vitro* e Transdução do Sinal, Departamento de Bioquímica, Instituto de Biologia, Universidade Estadual de Campinas (UNICAMP), Campinas, São Paulo, SP 13083-970, Brazil; E-Mails: dasilva.rodrigo.a@gmail.com (R.A.S); daisy.machado@gmail.com (D.M.); carmenv@unicamp.br (C.V.F.); 3 Intituto Federal de Educação, Ciência e Tecnologia do Rio Grande do Norte (IFRN), Santa Cruz, Rio Grande do Norte, RN 59200-000, Brazil; E-Mail: leandro-silva-costa@hotmail.com

**Keywords:** apoptosis, HeLa cells, maize, phenolic compounds

## Abstract

Neglected agricultural products (NAPs) are defined as discarded material in agricultural production. Corn cobs are a major waste of agriculture maize. Here, a methanolic extract from corn cobs (MEC) was obtained. MEC contains phenolic compounds, protein, carbohydrates (1.4:0.001:0.001). We evaluated the *in vitro* and *in vivo* antioxidant potential of MEC. Furthermore, its antiproliferative property against tumor cells was assessed through MTT assays and proteins related to apoptosis in tumor cells were examined by western blot. MEC showed no hydroxyl radical scavenger capacity, but it showed antioxidant activity in Total Antioxidant Capacity and DPPH scavenger ability assays. MEC showed higher Reducing Power than ascorbic acid and exhibited high Superoxide Scavenging activity. In tumor cell culture, MEC increased catalase, metallothionein and superoxide dismutase expression in accordance with the antioxidant tests. *In vivo* antioxidant test, MEC restored SOD and CAT, decreased malondialdehyde activities and showed high Trolox Equivalent Antioxidant Capacity in animals treated with CCl_4_. Furthermore, MEC decreased HeLa cells viability by apoptosis due an increase of Bax/Bcl-2 ratio, caspase 3 active. Protein kinase C expression increased was also detected in treated tumor cells. Thus, our findings pointed out the biotechnological potential of corn cobs as a source of molecules with pharmacological activity.

## 1. Introduction

Reactive species are molecules or atoms that have an electronic instability and for this reason have the characteristic of being highly reactive [1]. Consequently, reactive species can promote the oxidation of extracellular and intracellular biomolecules producing several damages to living organisms [2]. In order to protect their biomolecules from damage caused by reactive species, living organisms have developed antioxidant systems, which act by preventing or act directly blocking the formation of reactive species and prevent damage caused by these unstable molecules/atoms [3]. Human cells have used an enzymatic antioxidant system containing enzymes like superoxide dismutase (SOD), catalase (CT) and system containing small molecules (non-proteic), such as ascorbic acid, tocopherol, and glutathione [4]. These systems are critical for the redox status homeostasis and for protecting human cells. In fact, when this homeostasis is disrupted and there are more reactive species than antioxidants several diseases can develop. Actually, several reports have shown close associations between reactive species and human degenerative diseases like as aging, arthritis, malignant neoplasm and cardiovascular diseases [5].

In addition, human organisms have also used exogenous antioxidant mainly obtained from the food to protect their cells. Thus, in order to inhibit or reduce the effects of reactive species it is recommended to intake antioxidants and to add antioxidant in food products. Because of this, synthetic compounds, such as butylated hydroxyanisole (BHA), butylated hydroxytoluene (BHT), tert-butyl-hydroquinone (TBHQ) and propyl gallate (propyl 3,4,5-trihydroxybenzoate, PG) are widely used as antioxidants in the food industry [6]. However, these compounds have been regarded as toxic [7].

Due to possible problems associated with the use of synthetic antioxidants, the identification of novel antioxidants has been of great interest. In recent decades, several studies have reported that the extracts from various natural sources, mainly plants, posses antioxidant activity [8,9]. Moreover, many purified molecules from these sources have been reported as potential antioxidant compounds [10].

Furthermore, several studies have shown the relation between antioxidant and antiproliferative activities of compounds from plant sources [11,12]. Phytochemicals have been shown to inhibit the proliferation of different tumor cell lines, such as colon cancer cells HT29, breast cancer cells MCF-7 [13] and human liver cancer cells HepG2 [14,15]. The antitumor action of antioxidants seems to exhibit different mechanisms and specific reaction of each cell, but this generally will reduce the number of free radicals that can initiate the development of a tumor [16].

Many studies have reported the antioxidant and antiproliferative activities of extracts from plant sources. However, few studies have evaluated compounds from neglected agricultural products (NAPs). Included in the NAPs are sources like fruit peels (exocarp), endocarp, husk, wood, leaves that are not normally consumed by humans, crustacean exoskeletons, *etc*. In this context, the agriculture of maize produces various NAPs and the corn cobs can be described as one of the main ones. The maize production in 2010/2011 was approximately 810 million tons and estimates of corn cob production were approximately the same amount. However, only a small amount of corn cob produced has any iotechnological applications. For instance, corn cobs have been explored to develop novel cellulose fibers with similar properties to those of common textile fibers [17]. Besides, corn cobs are a promising biofuel fermentation substrate due to their low cost and high cellulose and hemicellulose content [18]. There is a great incentive to further exploit corn and its NAPs in order to provide a more complete characterization of its benefits. In this context, polysaccharides from corn cobs had been studied, they showed immunogenic [19], mitogenic [20], antioxidant and antiproliferative activities [21]. Moreover, the pharmacological potential of other molecules from corn cobs such as phenolic compounds had not been evaluated so far. Accordingly, the aim of this work was to obtain a methanolic polysaccharide free extract from corn cobs, and assesses its antioxidant activity using *in vitro* and *in vivo* tests *,* as well as, its effect on human adenocarcinoma cells (HeLa cells) viability.

## 2. Results and Discussion

### 2.1. MEC up-Regulates Antioxidant Activity in Vitro

Polar solvents such as ethanol, ethyl acetate and acetone, *etc.* are widely used for the extraction of antioxidant components from plant materials. However, extraction with methanol often results in a higher recovery of total extractable compounds [22]. Thus, we choose to work with methanolic extract. Due to its physico-chemical characteristics of high solubility of phenolic compounds in organic solvents, these molecules are probably involved in the antioxidant found in methanol extracts.

In the methanolic extract obtained in our work, the total phenolic, protein and carbohydrate content was 1.4:0.001:0.001 respectively, indicating that the observed biological activities are probably related to phenolic compounds due to its proportion in compared to proteins and carbohydrates. High amounts of phenolic compounds in methanolic extracts is very common, even in those obtained from NAPs like wood and pericarp of *Caesalpinia decapetala* [23].

Phenolic compounds are considered important antioxidants, so in our work we decided to evaluate the antioxidant potential of methanolic extract from corn cob (MEC), and for this purpose different antioxidant tests were used. Antioxidants are compounds that can prevent biological and chemical substances from radical-induced oxidation damage. Because radical oxidation of substrates occurs through a chain reaction involving three stages (*i.e.*, initiation, propagation and termination), antioxidants show their effects through various mechanisms. Thus, we used different methods to evaluate the effect of the corn cobs extract on initiation (total antioxidant capacity, DPPH assay, and power reducing), propagation (iron chelating) and termination (superoxide and hydroxyl radical scavenging activities) stages.

Initially, the MEC was evaluated in a test known as Total Antioxidant Capacity (TAC) [24]. The methanolic extract showed a TAC relative to 98.03 mg AAE (ascorbic acid equivalent)/g of sample. The material extracted with the solvent was fairly efficient in reducing the molybdenum in assay, indicating a high antioxidant potential of the sample. The TAC values obtained with MEC was similar to other natural extract [21,25], even when compared to another research that performed the TAC assay with methanolic extract [26].

**Figure 1:**
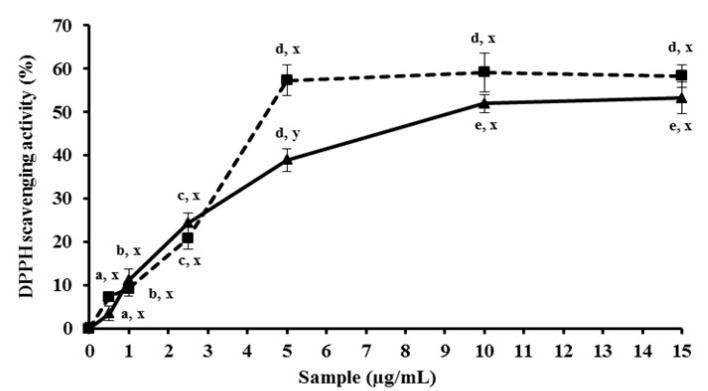
*In vitro* DPPH activity scavenging of MEC. The activity of Methanolic Extract is represented by the continuous line. The dashed line expresses activity of the known antioxidant α-tocopherol. MEC and positive control were used at the same concentrations (0; 0.5; 1.0; 2.5; 5.0; 10; 15 µg/mL). Letters ^a,b,c,d,e^ Indicate significantly differences between different concentrations of the same sample. ^x,y^ Represent significantly difference between different samples at similar concentrations. Student-Newman-Keuls test (*p* < 0.05).

The MEC also showed high DPPH radical scavenging activity; the maximum activity was achieved using about 10 µg/mL of sample, which reached a value of 50% of DPPH scavenging (Figure 1). DPPH scavenging results of MEC were similar to those of α-tocopherol—an established antioxidant compound—and only in high concentrations did this vitamin surpass the MEC activity (Figure 1). Li *et al.* [27] obtained hawthorn fruit extracts using different organic solvents and evaluated their antioxidant capacity. Compared to all extraction conditions of the cited work above, our methanol extract from corn cob reached a value 10 times higher than scavenging of DPPH.

In another study, the methanolic extract of corn cob showed an activity of about 16% of radical scavenging in the same DPPH assay [28]. The difference in results when compared to our data could be attributed to source properties. When the maize is subject to different periods of solar radiation, humidity, temperature and rainfall throughout the year, this can lead it to the synthesis of different proportions of molecules that will influence in extract activities.

The antioxidant proprieties of bran wheat extract were evaluated in another study and its free radical scavenging properties against DPPH were lower than those found for MEC [29]. The capacity to scavenge the DPPH radical also was measured with a methanolic extract from bran wheat, another NAP [30]. In this work, bran extracts from five wheat varieties indigenous to Pakistan were evaluated, and none of the samples showed the DPPH scavenging values higher than MEC.

**Figure 2:**
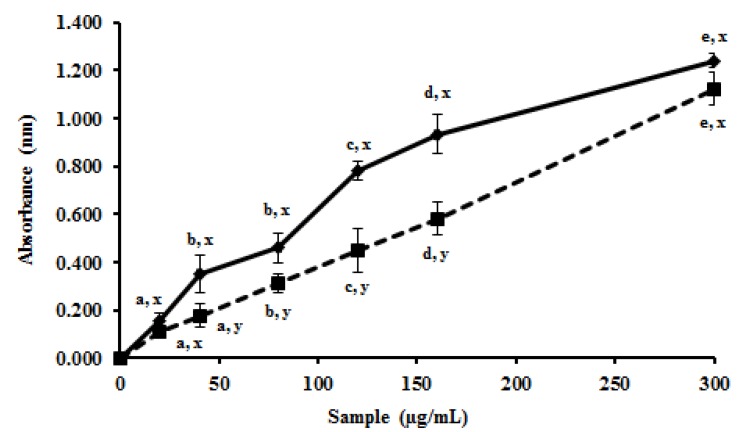
*In vitro* reducing power effect of MEC. The test expresses sample ability to reduce the iron ions Fe^3+^ to Fe^2+^. The activity of methanolic extract has been represented to continuous line. The dashed line expresses activity to already known antioxidant (ascorbic acid). MEC and ascorbic acid were used in the follow concentrations 0; 20; 40; 80; 120; 160; 300 µg/mL. ^a,b,c,d,e^ Indicate significantly differences between different concentrations of the same sample. ^x,y^ Represent significantly difference between different samples at similar concentrations. Student-Newman-Keuls test (*p* < 0.05) Absorbance at 700 nm.

In addition to the described tests, promising results were obtained in the reducing power assay. In summary, this method assesses the capacity of a sample to donate electrons in the presence of ferric chloride under acid conditions and thus reduce Fe^+3^ to Fe^+2^. The MEC showed absorbance values higher than ascorbic acid when we used the same mass to perform the test (Figure 2). The results obtained with MEC were higher than those obtained with other methanol extracts, such as those obtained with extract of *Armillaria mellea* that required a concentration five times higher than MEC to present the same activity [31]. This shows that MEC had a great electron-donator capacity to iron atoms in a slightly acidic environment, and then the extract could act as antioxidant in this condition.

We used two methods that evaluate the ability/capacity of a sample to donate electrons because we tried to simulate situations that may be found in living organisms. Since the chemical environment of each method is different, a molecule can exhibit good activity in one method but not in the other. According to the results of TAC and reducing power, the MEC had proton-donation ability and could serve as free radical inhibitors and act in the initiation stage of oxidation of cellular compounds, preventing certain molecules to be degraded in different environmental conditions found at the cells like as lysosome and mitochondria.

The MEC showed no activity under any of the conditions used for the test of iron chelating capacity and hydroxyl scavenging activity assay. The chelating ability of a compound is defined as the formation of bonds between two or more separate binding sites within the same molecule and a single central atom. This characteristic is usually attributed to organic compounds, such as polysaccharides, which bind to metal atoms form a chelate [32]. Analyzing thus, strong indications lead us to believe that absence of MEC activity in iron chelating capacity assay was due to the low amount of polysaccharides in the extract.

**Figure 3:**
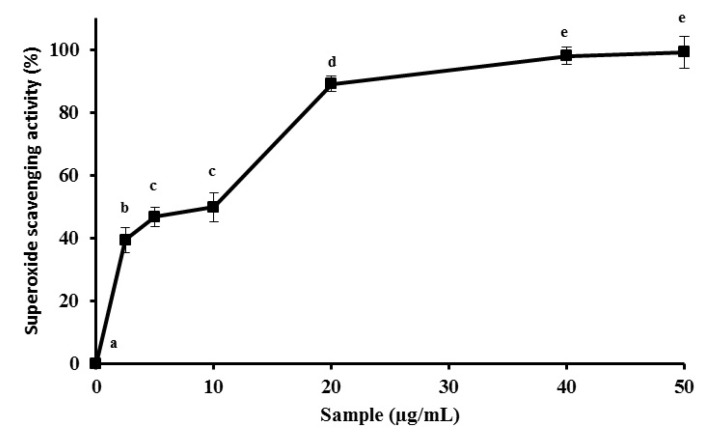
*In vitro* superoxide radicals scavenging activity of MEC. The methanolic extract reaches maximum activity with 40 µg/mL of sample. Sample concentrations used were 0; 2.5; 5.0; 10; 20; 40; 50 µg/mL. Letters ^a,b,c,d,e^ Indicate significantly differences between different concentrations of the same sample. Student-Newman-Keuls test (*p* < 0.05).

Some free radicals are formed in mitochondria as a result of the electron transport system carried out in this organelle [23]. The excess electrons can move out of this mitochondrial system and react with molecular oxygen to create many reactive oxygen species (ROS). Molecular oxygen with an additional electron, called superoxide anion, is extremely reactive and can promote oxidative degradation of lipids and important proteins which increases the probability of degenerative diseases, like Alzheimer's disease [33]. The data presented in this paper showed that MEC had substantial superoxide anion scavenging activity, with values of about 97.4% ± 3.7% activity being obtained with low amounts (40 µg/mL), an activity which remains constant with higher amounts of MEC (Figure 3). Other methanolic extracts showed high superoxide anion scavenging activity, such as the extracts from fruits and flowers of *Hypericum lydium* Boiss (around 100% of superoxide anion scavenging activity). However, the extract amount used to obtain this activity was much higher (10 mg/mL) [34] when compared to the MEC one used. In another work using NAPs, free radical and reactive oxygen species scavenging activities of peanut skin (exocarp) extract were measured; the maximum scavenging activity was achieved only when 500 µg/mL of sample was used, an activity value that is 10-fold lower than that of MEC [35]. Our results support the strong superoxide anion scavenging capacity of MEC. Overall, the antioxidant data obtained using MEC suggests a promising antioxidant potential of corn cobs. Furthermore, it can improve the utilization of corn cobs and reduce environmental pollution.

### 2.2. Effect of MEC on Cells Antioxidant Enzymes

Among the various causes of mutations in genetic material, DNA damage caused by increased oxidative stress appears as a crucial event to raise the number of significant changes in this important molecule. A large number of mutations can worsen the severity of tumor cells, can alter the behavior of these cells enabling them to invade nearby tissue or spread to organs distant from the site of origin. Tumor cells often have a weakened antioxidant system and repeatedly system failure occurs due to mutations in important genes involved in the repair of damaged molecules besides deficiency of enzymes and compounds to combat oxidative stress [36,37].

Since we have demonstrated that MEC displays antioxidant activity, this prompted us to investigate if a human cancer cell line (HeLa cells) treated with MEC would display any change in redox status. To do this, the expression of catalase (CAT), superoxide dismutase (SOD) and metallothionein (MT) in HeLa cells was checked.

The antioxidant enzyme system presents itself as an effective combatant of reactive oxygen species. Catalase is an important enzyme that catalyzes the decomposition of hydrogen peroxide to water and oxygen [38], while MT binds to metal atoms and decreases the production of hydroxyl radicals [39] and SOD catalyzes the dismutation of superoxide into oxygen and hydrogen peroxide [40].

**Figure 4:**
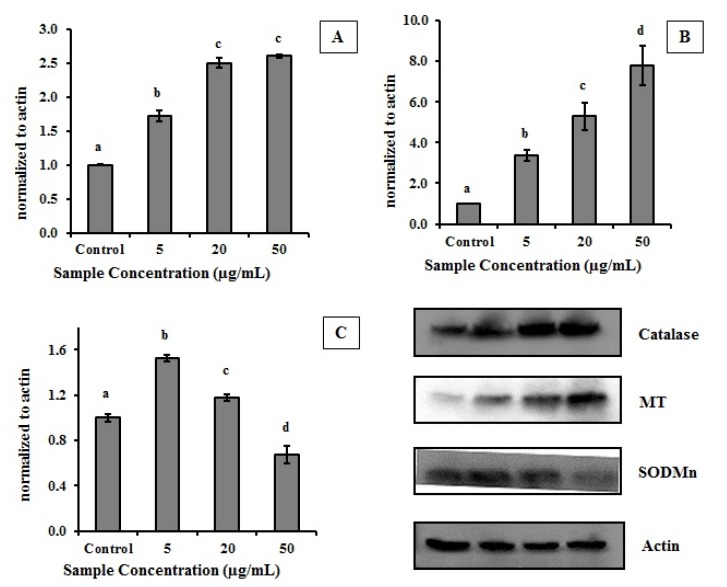
Effect of MEC on level of CAT, SOD and MT in HeLa cells. Graphic represents the relation of protein normalized to actin. (**A**) Catalase enzyme; (**B**) MT—Metallothionein; (**C**) SODMn—Superoxide dismutase. In the upper right are the images of the western blot of proteins involved. Letters ^a,b,c,d^ Indicate significantly differences between different concentrations of the same sample. Student-Newman-Keuls test (*p* < 0.05).

The treatment of HeLa cells with MEC resulted in changing the amount of antioxidant protein produced (Figure 4). CAT and MT expressions were higher in treated cells which indicated that MEC exerts antioxidant action in those cells by increasing intracellular production of key antioxidant enzymes.

CAT was increased about 2.5 fold in the presence of 20 µg/mL MEC (Figure 4A). MT increased about 8.0 fold when 50 µg/mL of the extract was used (Figure 4B). Another important enzyme involved in cellular antioxidant process is SOD; the amount of this enzyme was increased only 1.5-fold in the presence of MEC (5 µg/mL) compared to the control. However, the amount of enzyme decreases proportionally with the increase in the amount of extract used (Figure 4C).

### 2.3. In Vivo Antioxidant Potential of MEC

Since we have demonstrated that MEC displays antioxidant activity *in vitro* and in cell system, it prompted us to treat normal rats with MEC in order to detect any changes in their redox status. Thus, Trolox equivalent antioxidant capacity (TEAC) in serum and liver from CCl_4_-intoxicated rat were determined. According to results given in Table 1, vitamin E (positive control) and MEC produce an increase in TEAC values when compared to the CCl_4_ group. This increased antioxidant activity in samples following MEC administration could indicate a direct absorption of several antioxidant compounds.

**Table 1:** Effect of MEC on liver and serum Trolox equivalent antioxidant capacity (TEAC), MDA, CAT and SOD in CCL4-intoxicated rat.

Group	Liver †			
	TEAC	MDA	CAT	SOD
Control	1.00 ± 0.04	1.00 ± 0.04	1.00 ± 0.18	1.00 ± 0.16
CCl_4_	^a^ 0.65 ± 0.03	^a^ 2.85 ± 0.15	^a^ 0.50 ± 0.53	^a^ 0.70 ± 0.68
CCl_4_ + Vit E	^b^ 1.00 ± 0.05	^b^ 0.98 ± 0.07	^b^ 1.00 ± 0.18	^b^ 0.96 ± 0.13
CCl_4_ + MEC	^b^ 0.96 ± 0.04	^b^ 1.00 ± 0.07	^b^ 1.05 ± 0.13	^b^ 0.94 ± 0.93
**Group**	**Serum †**			
	**TEAC**	**MDA**	**CAT**	**SOD**
Control	1.00 ± 0.47	1.00 ± 0.7	1.00 ± 0.16	1.00 ± 0.84
CCl_4_	^a^ 0.60 ± 0.35	^a^ 2.26 ± 1.3	^a^ 0.78 ± 0.34	^a^ 0.60 ± 0.85
CCl_4_ + Vit E	^a^ 0.87 ± 0.55	^a,b^ 1.23 ± 0.9	^b^ 0.96 ± 0.39	^b^ 0.97 ± 0.76
CCl_4_ + MEC	^b^ 1.04 ± 0.36	^a,b^ 1.46 ± 0.7	^b^ 0.95 ± 0.40	^b^ 0.94 ± 0.11

† Data are expressed as values average ± standard deviation compared to control (*n* = 6 rats per group); Comparison between groups was made using the Student-Newman-Keuls test; ^a^ Significantly different from the control group (*p* < 0.05); ^b^ Significantly different from the CCL_4_ group (*p* < 0.05); SOD, superoxide dismutase; CAT, catalase; MDA, malondialdehyde; Vit E, vitamin E.

The SOD and CAT activities in liver and serum deceased with CCl_4_ treatment (Table 1) probably due the number of deleterious effects caused by the accumulation of trichloromethyl radicals (CCl_3_•) and trichloromethylperoxyl radicals (CCl_3_O_2_•), which appear in rats because CCl_4_ is metabolized in the cytocrome P450 system to give both reactive species. In addition, since SOD and CAT activities are low, CCl_3_O_2_• and other reactive species cause peroxidative degradation of cell lipid membranes, that leads to the formation of lipid peroxides, which produce malonaldehyde (MDA). MDA is one of the most important biomarker of lipid peroxidation. Thus, as expected, CCl_4_ increased the amount of MDA in the samples (Table 1).

MEC reestablished the levels of MDA even in the presence of CCl_4_, probably because MEC reestablished SOD and CAT activities. We showed that the presence of MEC in cell culture increased the level of CAT (Figure 4) which justifies in part the MEC effects *in vivo*. However, we also showed that MEC decreased the SOD amount in cells. In addition, we suggested that the SOD levels decrease in cells due the high MEC superoxide radical scavenging activity. Thus, we believe that MEC compounds responsible for MEC superoxide radicals scavenging activity were absorbed in low amounts or were not absorbed and/or they were quickly metabolized by rats. In addition, we suggest that MEC’s effects on antioxidant enzymes could be related to its ability to block the deleterious effects of reactive species resulting from the biotransformation of CCl_4_. However, further research is still needed to obtain more detailed information about the *in vivo* actions of MEC.

### 2.4. MEC Exhibits Anti-Proliferative and Pro-Apoptotic Activity

**Figure 5:**
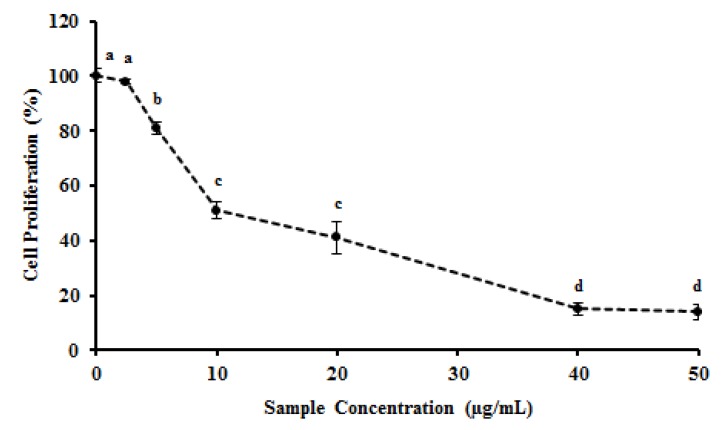
Effect of MEC on HeLa cell proliferation measured by MTT test. HeLa cells proliferation was carry out in the presence or absence of MEC (0; 2.5; 5.0; 10; 20; 40; 50 µg/mL). Letters ^a,b,c,d^ Indicate significantly differences between different concentrations of the same sample. Student-Newman-Keuls test (*p* < 0.05).

In addition to the antioxidant activity, other pharmacologic activities have been attributed to natural extracts from several sources such as the ability to inhibit or reduce the proliferation of tumor cells [41,42]. Thus, in order to investigate the potential of MEC as an antiproliferative agent, HeLa cells were treated with MEC and cellular viability assessed by MTT reduction. Figure 5 shows that MEC dramatically decreased HeLa cells viability. MEC showed IC_50_ for antiproliferative activity of 10 μg/mL with 80% inhibition of cell growth at 40 μg/mL. Quercetin, a phenolic compound used as positive control showed an IC_50_ of 1.5 μg/mL (data not shown).

In another study, methanolic extracts were used to assess the ability to inhibit tumor cell proliferation. An extract obtained from horse chestnut, which inhibited about 79% of HeLa cell proliferation at 125 µg/mL proved potent [43]. Ren *et al.* [44] used 80 µM of clitocine, a purified compound, which reduced HeLa cell viability about 90%, an antiproliferative effect similar to that shown by MEC results. Extract derived from olive oil by-products inhibited the proliferation of MDA-MB-231 breast cancer cells with 6 mg/mL of extract [45]. Thus, when we compared these data to the antiproliferative activity of MEC, we could infer that MEC had higher antitumor potential.

Additionally, to determine whether MEC (from 2.5 to 50 µg/mL) had unspecific cytotoxicity on any cell type, we also tested the effect of MEC on the growth of 3T3, a normal fibroblast cell line. With this cell line MEC showed inhibition of cell growth (20%) only at the highest dose (50 µg/mL; data not shown).

Next, we evaluated if the action of MEC as an antiproliferative agent is related to the induction of apoptosis. The cell death process involves several events closely related to dysfunction of mitochondrial and plasma membranes and the production of proteins involved in cell destruction. Apoptosis is a well-described mechanism of programmed cell death and an important event in the natural development of multicellular organisms. Compounds that induce tumor cell death by apoptosis are of great importance, since the apoptosis process does not involve cellular inflammatory cascades and therefore would not affect neighboring normal cells [46].

**Figure 6:**
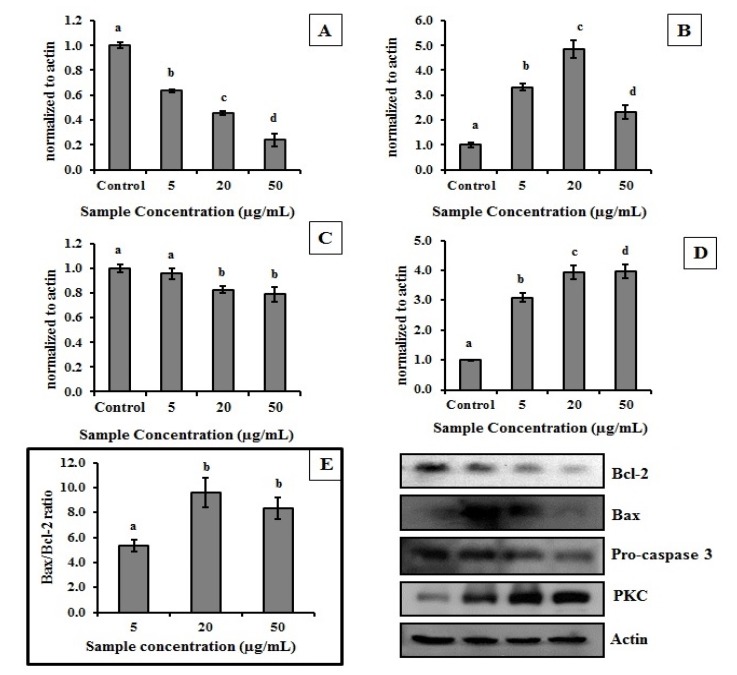
Effect of MEC on several proteins levels involved in cell death. Graphic represents the relation of protein normalized to actin. (**A**) Bcl-2 protein; (**B**) Bax protein; (**C**) pro-Caspase 3; (**D**) PKC – Protein kinase; (**E**) in the box, Bax/Bcl-2 ratio. In the upper right are the images of the western blot of proteins involved. Letters ^a,b,c,d^ Indicate significantly differences between different concentrations of the same sample. Student-Newman-Keuls test (*p* < 0.05).

Thus, in order to investigate if MEC induced apoptotic events in HeLa tumor cells, proteins related to cell death by apoptosis were analyzed. The Bax/Bcl-2 ratio is an important parameter to evaluate the apoptosis process [47]. The increased amount of Bax and decreased Bcl-2 will allow greater permeability of mitochondrial membrane due to the formation of pores by Bax family protein, and this fact will release factors involved in death cell [48]. HeLa tumor cells treated with MEC displayed an augment of the expression of Bax protein while Bcl-2 protein dropped (Figure 6).

Thus, Bax/Bcl-2 ratio has high values with a maximum of 9.6 when we used a concentration of 20 µg/mL of MEC (Figure 6F). Thus, we suggest that MEC antiproliferative mechanism is related to its capacity to induce the release of mitochondrial proteins involved in cell death.

The increase of apoptotic factors in the cytoplasm promotes the activation of enzymes closely related to apoptotic events, such as the cysteine-aspartic acid protease (caspase) family. Caspases exist as inactive proenzymes, termed pro-caspases, which undergo proteolytic processing producing subunits that dimerize to form the active enzyme [49]. Caspase-3 is activated in the apoptotic cell both by extrinsic (death ligand, usually in the surface of the cytoplasmic membrane) and intrinsic (mitochondrial, by the apoptossome complex) pathways [50]. According to the results obtained in the present study, the concentration of 20 µg/mL of MEC decreased the amount of pro-caspase 3 (Figure 5).

Besides those proteins mentioned here, other proteins and proteases have important role in the apoptosis process. Kinase protein families, such as protein kinase C (PKC) play central roles in cellular metabolism. In our study, it was observed that HeLa cells treated with 20 µg/mL MEC displayed a significant increase (around 4-fold) in their PKC expression. Therefore, this kinase may play a crucial role in the mechanism of action of MEC. However, further studies are needed to confirm this hypothesis.

## 3. Experimental

### 3.1. Materials

Potassium ferricyanide, ferrous sulfate (II), n-propanol, acetic acid, ethanol, and sulfuric acid were obtained from Merck (Darmstadt, Germany). Sodium chloride was purchased from Sigma Chemical Co. (St. Louis, MO, USA). HeLa cells (ATCC CCL-2) were a gift from Silvia R. B. Medeiros, Department of Genetic and Cell Biology, UFRN, Brazil. Embryo fibroblast 3T3 (ATCC CCL-164) were a gift from Carmen Ferreira (Department of Biochemistry, UNICAMP, Campinas, SP, Brazil). Cell culture medium components (Dulbecco’s-Minimum essential medium (DMEM)), L-glutamine, sodium bicarbonate, non-essential amino acids, sodium pyruvate, fetal bovine serum (FBS), and phosphate buffered saline (PBS) were purchased from Invitrogen Corporation (Burlington, ON, Canada). All other solvents and chemicals were of analytical grade.

### 3.2. Production of Methanolic Extract from Corn Cob (MEC)

Fresh corn samples, purchased at a local market, were cleaned, washed, and grains were completely stripped to avoid contamination of the seeds. They were further chopped into small pieces, dried, and grounded into flour. A methanol (160 mL) was added to 10 g of corn cob powder and stirred in the dark and at 22 °C for about 24 h. After that, the solvent was evaporated and permanent mass (~1.7 g) kept protected from light and used for further analysis.

### 3.3. In Vitro Antioxidant Tests

#### 3.3.1. Total Antioxidant Capacity (TAC)

The method of assay is based on the reduction of molybdenum VI (Mo^+6^) to Mo^+5^ by the sample and subsequent formation of a green phosphate/Mo^+5^ complex in an acidic solution. The samples were incubated at 95 °C for 90 min; the tubes contained the MEC and reagent solution (0.6 M sulfuric acid, 28 mM sodium phosphate and 4 mM ammonium molybdate). After the mixture had cooled to room temperature, the absorbance of each solution was measured at 695 nm against a blank. The antioxidant capacity was expressed as mg of ascorbic acid equivalent/g of sample.

#### 3.3.2. The Hydroxyl Radical Scavenging Activity

The hydroxyl radical scavenging activity of MEC was investigated using Fenton’s reaction as the assay basis. The results were expressed as the rate of inhibition. Hydroxyl radicals were generated using a previously described method in 3 mL of 150 mM sodium phosphate buffer (pH 7.4) containing 10 mM FeSO_4_ × 7H_2_O, 10 mM EDTA, 2 mM sodium salicylate, 30% H_2_O_2_ and to assess the activity of sample different amount of MEC (0; 20; 40; 80; 120; 160; 300 µg/mL) were used. In the control, sodium phosphate buffer replaced the H_2_O_2_. The solutions were incubated at 37 °C for 1 h, and monitoring absorbance at 510 nm.

#### 3.3.3. The Superoxide Radical Scavenging Activity

The superoxide radical scavenging assay was based on the capacity of MEC (0; 2.5; 5.0; 10; 20; 40; 50 µg/mL) to inhibit the photochemical reduction of nitroblue tetrazolium (NBT) in the riboflavin-light-NBT system. Each 3 mL reaction mixture contained 50 mM phosphate buffer (pH 7.8), 13 mM methionine, 2 mM riboflavin, 100 mM EDTA, 75 mM NBT, and 1 mL sample solution. After 10 min of illumination with a fluorescent light source, the production of blue formazan was monitored as absorbance increased at 560 nm. The entire reaction assembly was enclosed in a box lined with aluminum foil. Identical tubes with the reaction mixture were kept in the dark and served as the blank.

#### 3.3.4. Ferric Chelating

The ferrous ion chelating ability of samples was investigated according previously described [51]. Briefly, the reaction mixture that contained samples (0; 20; 40; 80; 120; 160; 300 µg/mL), FeCl_2_ (0.05 mL, 2 mM) and ferrozine (0.2 mL, 5 mM) was mixed well and incubated for 10 min at room temperature. The absorbance of the mixture was measured at 562 nm against a blank.

#### 3.3.5. Reducing Power

Briefly, 4 mL of reaction mixture containing different MEC or ascorbic acid concentrations (0; 20; 40; 80; 120; 160; 300 µg/mL) in 0.2 M phosphate buffer (pH 6.6), was incubated with potassium ferricyanide (1% *w/v*) at 50 °C for 20 min. The reaction was stopped by addition of TCA solution (10% *w/v*). The solution was then mixed with distilled water and ferric chloride (0.1% *w/v*) solution, and the absorbance was measured at 700 nm.

#### 3.3.6. DPPH Assay

The hydrogen-donating or radical scavenging ability to stable DPPH radical in a methanolic solution was measured in this assay. Methanolic solution of DPPH (3 mL, 4 × 10^−6^ mol/L) was added to 1 mL of MEC or positive control (0; 0.5; 1; 2.5; 5; 10; 15 µg/mL) dissolved in methanol [52]. The decrease in absorbance at 517 nm was determined after 30 min in the darkness.

### 3.4. In Vivo Antioxidant Tests

Wistar rats (200–220 g) were used in the course of this study. After an acclimation period, the rats were randomly divided into four groups of six each. The first group (untreated control) received saline (0.9%, *w/v*) daily by oral gavage. Rats of second receive CCl_4_: oil mineral (1:1, 2 mL/kg bw/day, s.c.). The third group received a daily oral dose of vitamin E (50 mg/kg bodyweight). The fourth group was given daily oral dose of MEC in saline solution (100 mg/Kg bw). In addition, the rats of third and fourth groups were administered simultaneously with CCl_4_: oil mineral (1:1, 2 mL/kg bw/day, s.c.) on alternate days after 30 min of MEC or vitamin E administration. 24 h after the last CCl_4_ administration, in 21th day, the animals were euthanized with 20 mg/Kg thiopental (0.5 g Thiopenthax, Cristália, São Paulo, Brazil). Their blood samples were collected by cardiac puncture in tubes contained citrate and centrifuged (3000 *g*, 15 min, 4 °C) and the plasma was keep at −20 °C for further analysis. The livers of rats were dissected immediately their death, frozen in dry ice and stored at −80 °C until use for analysis.

#### 3.4.1. Determination of Total Antioxidant Capacity of Serum and Liver Homogenate

In order to determinate the antioxidant capacity of samples from rats we performed a Trolox equivalent antioxidant capacity (TEAC) assay as described before [53]. Briefly, 7 mM 2,20-azinobis-(3-ethyl-benzothiazoline-6-sulfonate) (ABTS, 5 mL) was added to 140 mM potassium persulfate (0.088 mL). After 12 h in dark (25 °C) this solution was diluted with ethanol (98%) to an absorbance of 0.7 ± 0.05 at 734 nm. Two mL of this diluted ABTS solution was added to samples (0.02 mL). The absorbance was measured 6 min after at 734 nm. TEAC was expressed as µM Trolox equivalent per mg protein.

#### 3.4.2. Malonaldehyde (MDA) Levels

To assess lipid peroxidation, MDA production was measured with the thiobarbituric acid reaction. Liver, about 1 mg, was homogenized in ice cold 0.15 M KCl solution (9 mL) and centrifuged (10,000 g, 20 min, 4 °C). An aliquot of the resulting supernatant (0.25 mL) was added to thiobarbituric acid solution (1.5 mL of 1% H_3_PO_4_ and 0.5 mL of 0.6% thiobarbituric acid). After 45 min at 100 °C in a water bath, *n*-butanol (2 mL) was added and the samples were stirred and centrifuged (10,000 g, 4 °C, 15 min). The butane layer absorbance was measured at 520 nm (L1) and 533 nm (L2) (Genesys 10s, UV-Vis, Thermo Scientific, Madison, WI, USA). The MDA amount was determined as L2-L1 and expressed as nmol/g of liver tissue.

#### 3.4.3. Catalase Activity

The catalase activity from rat liver extract was measured according to the Aebi method [54]. The disappearance of hydrogen peroxide was observed using a spectrophotometer (240 nm, 1 min, 25° C). The enzyme activity was determined using an extinction coefficient of 0.043/mM/cm^−1^. One unit of activity corresponds to the mmol of H_2_O_2_ destroyed/min/mg protein.

#### 3.4.4. Superoxide Dismutase (SOD) Activity

SOD activity from rat liver extract was assayed spectrophotometrically as described [55]. One SOD unit represents the amount of enzymes required to inhibit the rate of NBT oxidation by 50%. The activity was expressed as unit/mg of protein.

### 3.5. MTT Assay

HeLa cells were grown in culture flasks in DMEM medium with 10% fetal bovine serium. Cells were plated into 96-well plates at a density of 5 × 10^3^ cell/well and allowed to attach for overnight at 37 °C and 5% CO_2_. In the antiproliferative assay, MEC was added (0; 2.5; 5.0; 10; 20; 40; 50 µg/mL). After 72 h incubation, traces of extract were removed by washing the cells with PBS and fresh medium and 10 μL of 12 mM 3-(4,5-dimethylthiazol-2-yl)-2,5-diphenyltetrazolium bromide (MTT) dissolved in PBS was added to determine the effects of the sample on cell proliferation. The cells were then incubated for 4 h at 37 °C and 5% CO_2_. To solubilize the reduced MTT product, isopropanol (100 μL) containing 0.04 N HCl was added to each well and thoroughly mixed using a multichannel pipettor. Within 1 h of HCl-isopropanol addition, the absorbance at 570 nm was read using a Multiskan Ascent Microplate Reader (Thermo Labsystems, Franklin, MA, USA). The percent of cell proliferation was calculated as follows:

Cell proliferation (%) = 
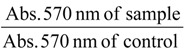
 × 100
(1)


### 3.6. Western Blot (Immunoblot)

The western blot technique was employed to evaluate the influence of the extract in the amount of proteins related to the enzymatic antioxidant system and apoptotic cell death. Briefly, 2 × 10^5^ cell/well was cultured in the six-well plates. MEC was added to the culture according to the required test. After treatment, cells were collected and then lysed with lysis buffer (50 mM Tris-HCl, pH 7.5, 150 mM NaCl, 1% Nonidet P-40, 2 mM EDTA, 1 mM EGTA, 1 mM NaVO_3_, 10 mMNaF, 1 mM DTT, 1 mM PMSF, 25 μg/mL aprotinin, and 25 μg/mL leupeptin) and kept on ice. The lysates were then centrifuged at 12,000 *×g* at 4 °C for 20 min; the supernatants were stored at −70 °C until use. The protein concentration was determined using the Bradford method. Aliquots of the lysates were separated by 12% SDS-PAGE and transferred to a nitrocellulose membrane using transfer buffer (192 mM glycine, 25 mM Tris-HCl, pH 8.8, and 20% methanol (*v/v*)). After blocking with 5% nonfat dried milk, the membrane was incubated for 2 h with primary antibodies, and then by 30 min with secondary antibodies in milk-containing tris-buffered saline (TBS) and 0.5% Tween. Anti-human Procaspase-3, Bcl-2, Bax, PKC, Catalase, MT, SODMn and β-Actin antibodies (Cell Signaling Technology, Inc., Danvers, MA, USA) were used at a 1:1000 dilution as the primary antibodies, while horseradish peroxidase-conjugated horse anti-rabbit IgG (Sigma Chemicals, St. Louis, MO,USA) was used at a 1:5,000 dilution as the secondary antibody. The membrane was then exposed, and protein bands were detected using enhanced chemiluminescence. All chemicals used were of research grade.

### 3.7. Statistical Analysis

All data are expressed as means ± standard of quadruplicates measurements. Each experiment was performed at least three times. Statistical analysis was performed by one-way ANOVA using the SPSS Statistic version 17.0-2008 software. Student-Newmans-Keuls post-tests were performed for multiple group comparison. In all cases, statistical significance was set at *p* < 0.05.

## 4. Conclusions

The methanolic extract from corn cob powder displays potential pharmacologic activities. MEC showed *in vitro* and *in vivo* antioxidant capacity. High values in the total antioxidant capacity, DPPH assay, reducing power and scavenging of superoxide radicals demonstrated that the extract has powerful antioxidant activity, probably due to the amount of phenolic compounds in the extract. MEC restored the levels of antioxidant enzymes (SOD and CAT) and the TEAC values, moreover, it showed a protective effect by reducing the production of MDA in animals treated with CCl_4_. In HeLa cell culture, MEC culture increased the production of protein related with the cellular enzymatic antioxidant system. Furthermore, MEC also showed antiproliferative activity against cancer cells, and its possible mechanism of action may be related to the change in the amount of proteins involved in the cell death process. Thus, our results suggest a high biotechnological potential for corn cob and reveals this agricultural by-product to be a source of molecules with pharmacological activity.
